# An optimised protocol for platelet-rich plasma preparation to improve its angiogenic and regenerative properties

**DOI:** 10.1038/s41598-018-19419-6

**Published:** 2018-01-24

**Authors:** Julia Etulain, Hebe A. Mena, Roberto P. Meiss, Gustavo Frechtel, Susana Gutt, Soledad Negrotto, Mirta Schattner

**Affiliations:** 1 Laboratory of Experimental Thrombosis, Institute of Experimental Medicine, CONICET-National Academy of Medicine, Buenos Aires, Argentina; 2 Division Experimental Pathology, National Academy of Medicine, Buenos Aires, Argentina; 3 Genetics and Molecular Biology, Department of Microbiology, Immunology and Biotechnology, School of Pharmacy and Biochemistry, University of Buenos Aires (UBA), Buenos Aires, Argentina; 4 Nutrition Service, Hospital Italiano, Buenos Aires, Argentina

## Abstract

Although platelet-rich plasma (PRP) is used as a source of growth factors in regenerative medicine, its effectiveness remains controversial, partially due to the absence of PRP preparation protocols based on the regenerative role of platelets. Here, we aimed to optimise the protocol by analysing PRP angiogenic and regenerative properties. Three optimising strategies were evaluated: dilution, 4 °C pre-incubation, and plasma cryoprecipitate supplementation. Following coagulation, PRP releasates (PRPr) were used to induce angiogenesis *in vitro* (HMEC-1 proliferation, migration, and tubule formation) and *in vivo* (chorioallantoic membrane), as well as regeneration of excisional wounds on mouse skin. Washed platelet releasates induced greater angiogenesis than PRPr due to the anti-angiogenic effect of plasma, which was decreased by diluting PRPr with saline. Angiogenesis was also improved by both PRP pre-incubation at 4 °C and cryoprecipitate supplementation. A combination of optimising variables exerted an additive effect, thereby increasing the angiogenic activity of PRPr from healthy donors and diabetic patients. Optimised PRPr induced faster and more efficient mouse skin wound repair compared to that induced by non-optimised PRPr. Acetylsalicylic acid inhibited angiogenesis and tissue regeneration mediated by PRPr; this inhibition was reversed following optimisation. Our findings indicate that PRP pre-incubation at 4 °C, PRPr dilution, and cryoprecipitate supplementation improve the angiogenic and regenerative properties of PRP compared to the obtained by current methods.

## Introduction

Wound repair is a dynamic and physiological process for regenerating damaged tissues^1^. Physiological wound healing may be disrupted by local factors (foreign bodies at the wound site, tissue maceration, ischaemia, or infection) or intrinsic individual factors (age, inflammatory diseases, drugs, or malnutrition) resulting in several clinical complications, including abnormal scarring, pain, pruritus, malignant transformation (Marjolin’s ulcer), haemorrhage, ulcer, infection, and amputation. These complications affect morbidity and mortality rates; hence, the healing of wounds is a current medical challenge^2^. Currently there are several techniques to promote wound repair, including biological tissue replacement, gene therapy, recombinant growth factors, and cell-based treatments^3,4^. Additionally, there are local methods for improving blood circulation in patients with chronic wounds associated with neuropathies and vascular diseases. These techniques include mechanical/physical methods (negative pressure therapy and intermittent pneumatic compression injuries) and ionic methods (hyperbaric treatment with ozone)^4^.

Platelet-rich plasma (PRP) constitutes an alternative therapy to promote tissue regeneration mediated by the release of several growth factors and cytokines stored in the alpha granules of platelets. These molecules modulate angiogenesis, remodel the extracellular matrix and affect the recruitment, proliferation, and differentiation of stem cells^5,6^. In contrast with other regenerative therapies, generating PRP is an economical method and does not require complex equipment or training. In addition, due to its primarily autologous origin and relatively non-invasive collection technique, the risks of infection or immune rejection associated with PRP are minimised^5,6^.

PRP is used in regenerative medicine for treating several clinical conditions, including ulcers, burns, muscle damage, and bone diseases and in tissue recovery after surgery. Despite the large variability of applications, the efficacy of regenerative treatments using PRP has been called into question due to the absence of large controlled clinical trials and consensus regarding PRP preparation techniques. The procedures currently used are derived from classical protocols for obtaining platelet concentrates for transfusion, coagulation assays or platelet functionality, which are focused on preserving platelet haemostatic but not regenerative responses. Thus, in this study, we aimed to optimise the protocol for PRP preparation, attempting to maximise the healing properties of platelets and analysing the effects on angiogenic and tissue regenerative responses.

## Results

### Plasma inhibits angiogenesis mediated by platelet-derived growth factors

**Figure 1:**
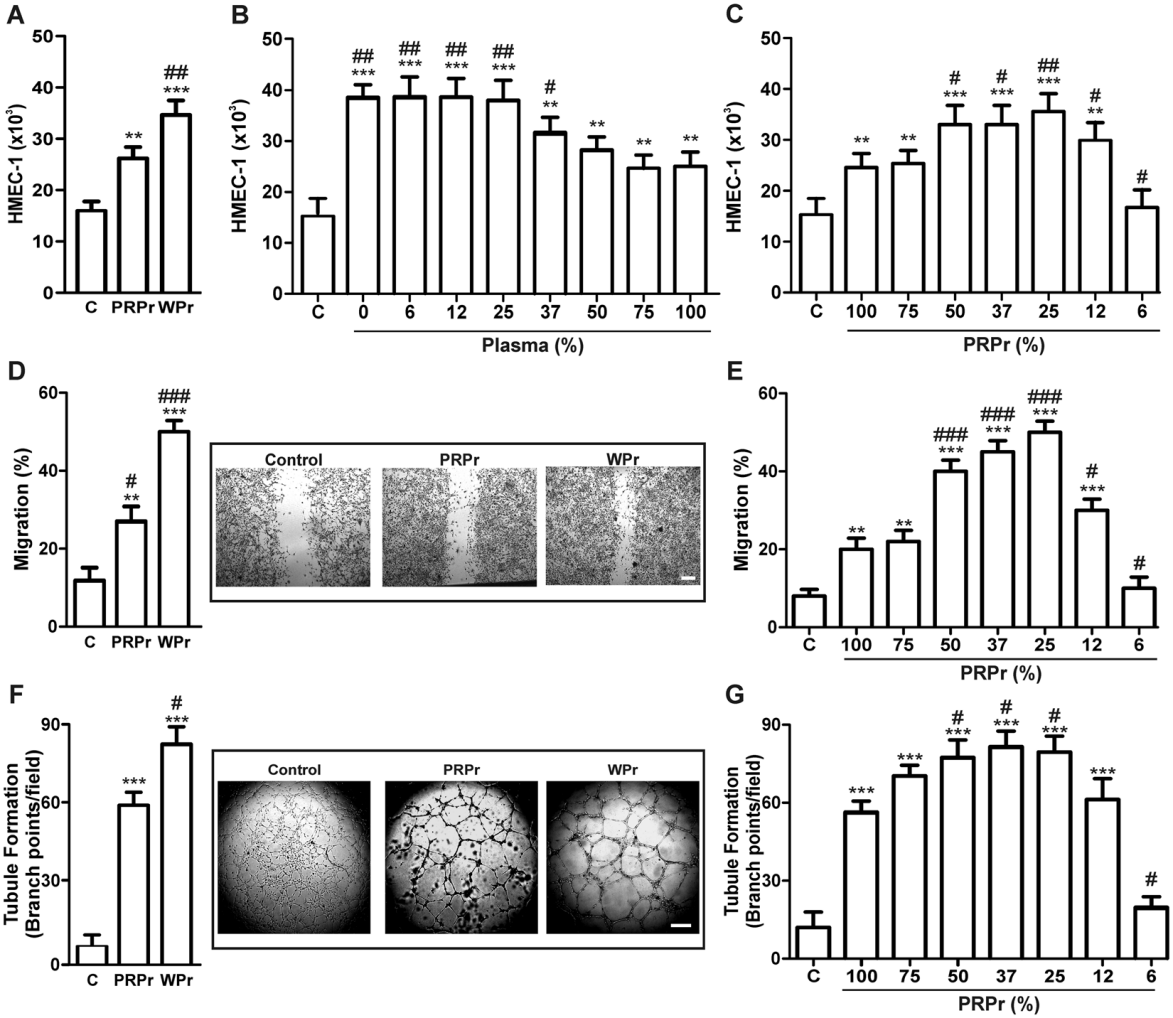
Plasma inhibits angiogenesis mediated by platelets. (**A**) HMEC-1 (15 × 10^3^) were incubated with saline plus FBS 2% (control), PRPr or WPr; (**B**) WPr supplemented with increasing % of plasma; or (**C**) PRPr diluted with saline, and endothelial cell proliferation was determined after 24 h by acid phosphatase activity assay. (**D,E**) Migration into scratched monolayers of endothelial cells and (**F,G**) tubule formation in Matrigel-coated wells was induced, as indicated, with WPr and PRPr (diluted or not). Images are representative of four to seven independent experiments (magnification 40X, scale bars: 200 μm). (n = 4–7; **P < 0.01, ***P < 0.001 vs. control; ^#^P < 0.05, ^##^P < 0.01, ^###^P < 0.001 vs. PRP 100%).

It is well-known that platelet-derived growth factors promote angiogenesis, a crucial initial step for tissue regeneration mediated by PRP. However, our previous findings regarding the release of pro- and anti-angiogenic molecules following activation of thrombin receptors (PAR-1 or PAR-4) suggested that plasma interferes with the pro-angiogenic role of platelets^7^. Surprisingly, a possible contribution of the high levels of physiological anti-angiogenic molecules present in plasma^7,8^ has not been considered in the context of PRP in tissue regeneration. To evaluate whether plasma interferes with the pro-angiogenic role of platelets, we first compared angiogenesis induced by platelets in the presence or absence of plasma. For these experiments, releasates obtained following PRP coagulation (PRPr) or plasma-free activated platelets (washed platelets, WPr) were used to induce endothelial proliferation, migration, and tubule formation in a human microvascular endothelial cell line (HMEC-1). Although the platelet concentration in WP and PRP was similar (450 ± 51 × 10^9^ and 453 ± 57 × 10^9^ platelets/L, respectively), endothelial proliferation induced by WPr was significantly higher (P < 0.01) than that induced by PRPr (Fig. 1A). Furthermore, when endothelial proliferation was induced by WPr in the presence of increasing amounts of plasma, HMEC-1 proliferation was proportionally inhibited by the increasing plasma concentration (Fig. 1B). This phenomenon was not associated with a cytotoxic effect because necrotic or apoptotic cells never exceeded 1% in any of the plasma treatments (data not shown).

To find the optimal balance between the opposite mitogenic effects of platelets and plasma, PRPr were diluted with increasing amounts of saline solution, and HMEC-1 proliferation was determined 24 h later. As shown in Fig. 1C, dilution improved the ability of PRPr to induce HMEC-1 growth, with a maximum peak at 25% PRPr; at higher dilutions, the effect decreased until it was completely lost at 6% PRPr. Similar results were obtained for HMEC-1 migration (Fig. 1D,E) and tubule formation (Fig. 1F,G). Therefore, the concentration of 25% PRPr was selected for further experimentation.

### Cold preconditioning, plasma cryoprecipitate supplementation, and dilution enhance angiogenesis mediated by PRPr

**Figure 2:**
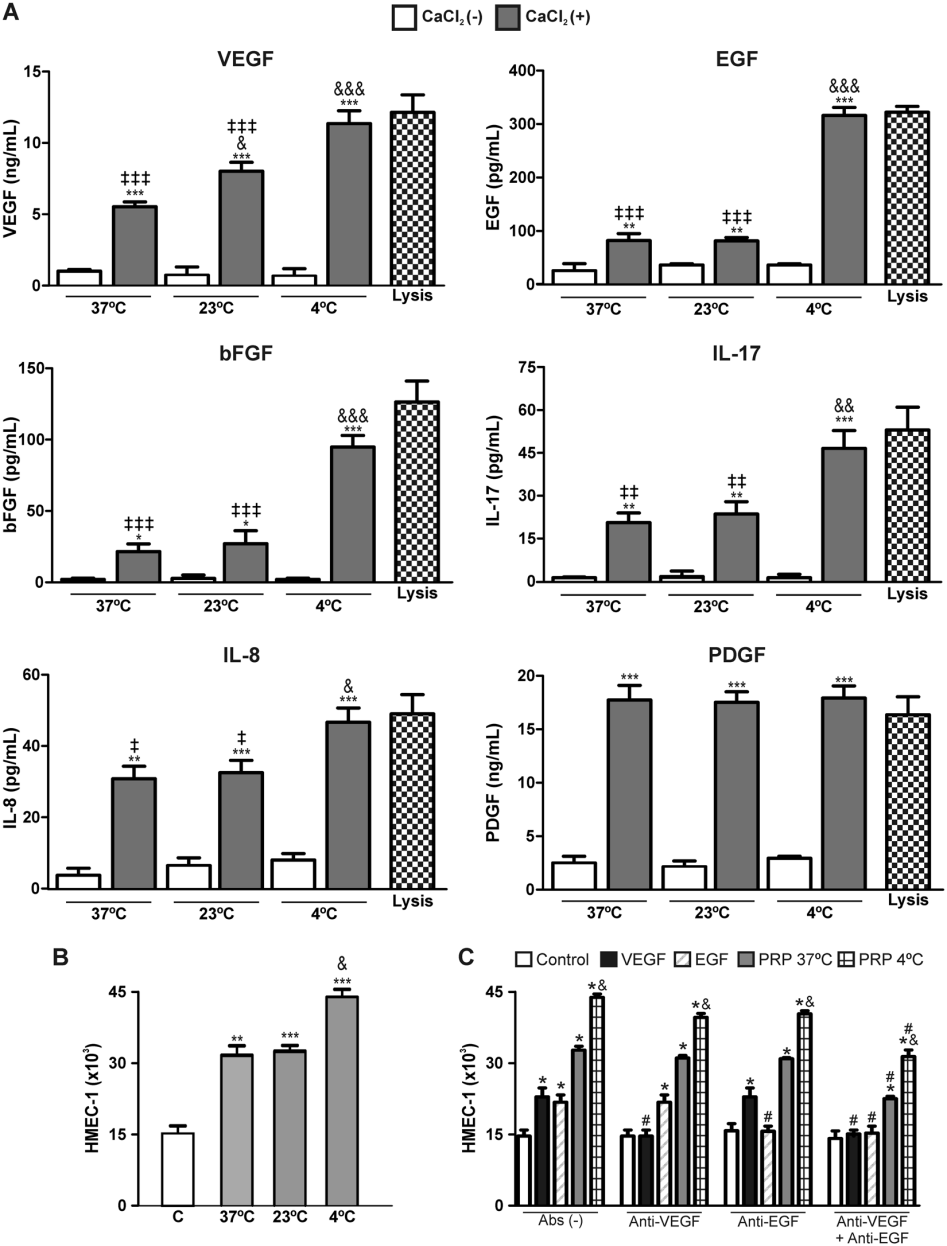
Cold preconditioning promotes the release of growth factors and cytokines from platelets. (**A**) PRP was incubated at 37 °C, 23 °C, or 4 °C for 30 min, and the levels of VEGF, EGF, bFGF, IL-17, IL-8, and PDGF in releasates of PRP before clotting (CaCl_2_−) or after clotting (CaCl_2_+) were determined by ELISA. Total intra-platelet levels were measured in platelet lysates. (**B**) PRP was incubated at 37 °C, 23 °C, or 4 °C for 30 min, clotted with CaCl_2_ (22 mM) for 40 min, and releasates were used to induce HMEC-1 (15 × 10^3^) proliferation. Saline plus FBS 2% was used as control. (**C**) HMEC-1 (15 × 10^3^) were incubated, individually or in combinations, with anti-human VEGFR2/KDR/Flk-1 (10 µg/ml), anti-human-EGFR (40 µg/ml) or irrelevant IgG, for 30 min. Next, endothelial proliferation was induced by addition of recombinant VEGF (20 ng/ml), EGF (20 ng/ml) or with PRPr preincubated at 37 °C or 4 °C and then clotting was induced with CaCl_2_ (22 mM). Saline plus FBS 2% was used as control (n = 4–5, *P < 0.05, **P < 0.01, ***P < 0.001 vs. unstimulated; ^&^P < 0.05, ^&&^P < 0.01, ^&&&^P < 0.001 vs. 37 °C; ^‡^P < 0.05, ^‡‡^P < 0.01, ^‡‡‡^P < 0.001 vs. lysis; ^#^P < 0.05 vs. without neutralizing antibodies (Abs)). VEGF: vascular endothelial growth factor; EGF: epidermal growth factor; bFGF: basic fibroblast growth factor; PDGF: platelet derived growth factor; IL: interleukin.

Although PRP is currently processed at 37 °C or room temperature (RT) (20–25 °C), it is well-known that low temperatures enhance platelet activation, improving the release of dense and alpha granules^9^. Thus, we evaluated the effect of cold preconditioning on the release of platelet-derived growth factors. For this purpose, PRP was incubated at 37 °C, 23 °C, or 4 °C for 30 min before the generation of PRPr. Next, levels of several pro-angiogenic molecules, such as vascular endothelial growth factor (VEGF), platelet derived growth factor (PDGF), epidermal growth factor (EGF), basic fibroblast growth factor (bFGF), interleukin (IL)-17, and IL-8 were determined in PRPr and compared to the total amount of each protein in platelet lysates. As shown in Fig. 2A, the secretion of VEGF, EGF, bFGF, IL-17, and IL-8, but not PDGF, was induced in a temperature-dependent manner. While VEGF, EGF, bFGF, IL-17, and IL-8 were partially released when PRP was incubated at 37 °C or 23 °C (20–60% of the total intra-platelet amount), total secretion of these molecules was only achieved when PRP was incubated at 4 °C, indicating that cold preconditioning maximises the release of platelet-derived pro-angiogenic molecules. In agreement with these results, proliferation of HMEC-1 triggered by PRPr obtained from PRP preconditioned at 4 °C was increased compared to that induced by PRP preconditioned at 37 °C (Fig. 2B). In addition, we observed that phosphorylation of p38–a relevant process involved in granule secretion from platelets–was increased as a result of exposure of platelets to 4 °C (Suppl. Fig. 1A). Moreover, a complete reversion of the enhanced angiogenesis mediated by PRP cold preconditioning was observed when platelet p38 phosphorylation was blocked by the specific inhibitor, SB203580, indicating that cold preconditioning primes platelet degranulation by enhancing activation of p38-mediated signalling (Suppl. Fig. 1B).

To further identify the relevant mediator(s) responsible for the proangiogenic activity of cold preconditioning, the contribution of VEGF and EGF (as the major factors released following 4 °C preconditioning) was evaluated by inducing endothelial proliferation in the presence of VEGF and EGF receptor-neutralizing antibodies. Our findings demonstrate that combined but not individual blockade of both molecules resulted in a moderate inhibition of endothelial proliferation (17% inhibition) in PRPr at 37 °C and 4 °C, without altering the ratio of angiogenesis observed at these temperatures (Fig. 2C).

**Figure 3:**
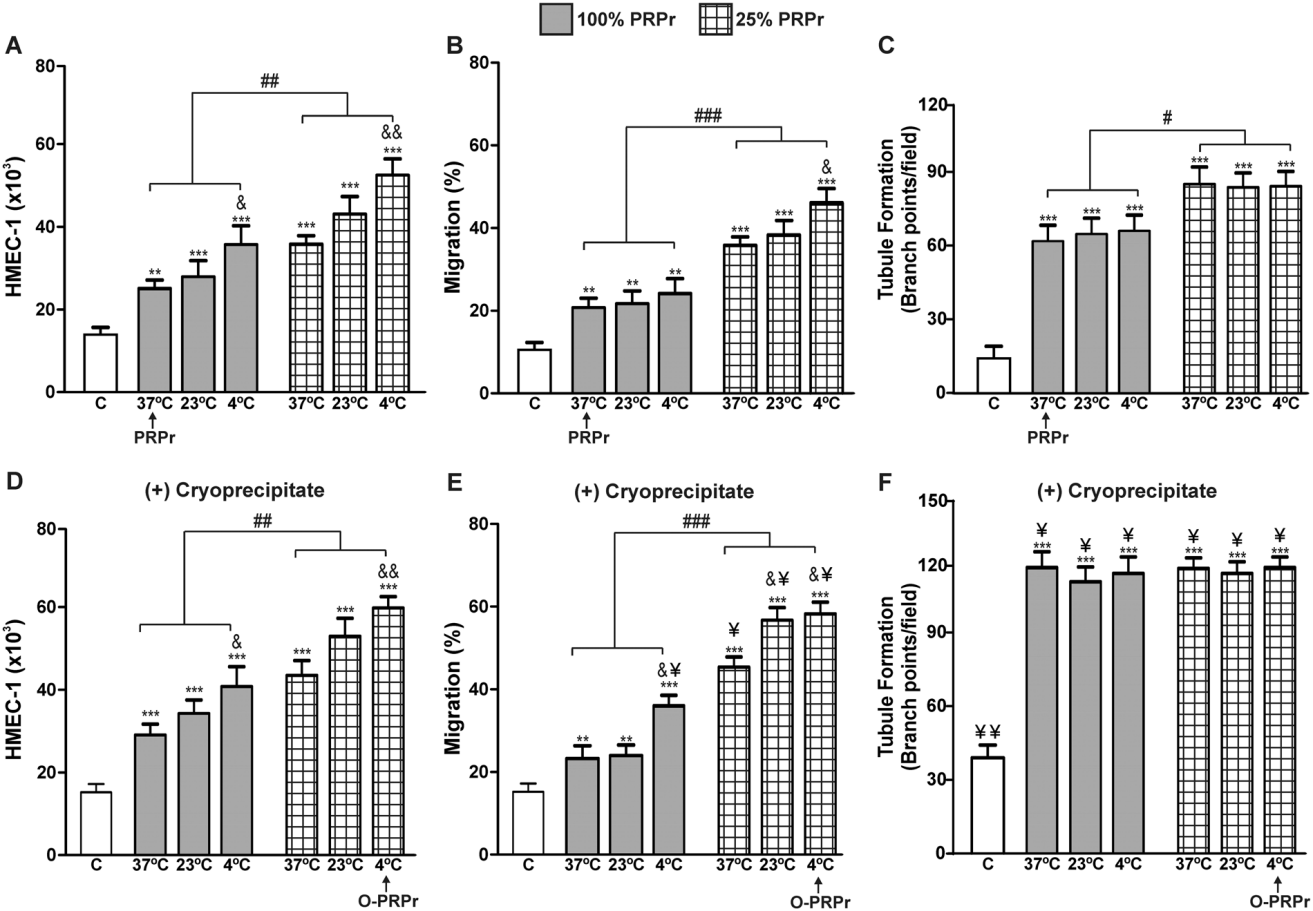
PRP cold preconditioning, PRPr dilution, and plasma cryoprecipitate supplementation enhance angiogenesis mediated by PRP. PRP was incubated at 37 °C, 23 °C, or 4 °C for 30 min and then clotted by the addition of CaCl_2_ (22 mM) for 40 min. HMEC-1 (15 × 10^3^) were incubated with PRPr pure (100%) or diluted with saline (25%). Saline containing 2% FBS was used as the control. Endothelial proliferation, migration, and tubule formation were determined *in vitro* without (**A–C**) or with plasma cryoprecipitate supplementation (**D–F**). (n = 5; **P < 0.01, ***P < 0.001 vs. control; ^#^P < 0.05, ^##^P < 0.01, ^###^P < 0.001 vs. PRP 100%; ^&^P < 0.05; ^&&^P < 0.01 vs. 37 °C; ^¥^P < 0.05 vs. without plasma cryoprecipitate).

Interestingly, an additive effect was observed by the combination of 4 °C PRP preconditioning and PRPr dilution, with a significant improvement in HMEC-1 proliferation compared to that induced by non-optimised PRPr (Fig. 3A). When the effect of temperature was analysed on HMEC-1 migration, no differences were observed between 37 °C and 4 °C. However, the effects of PRPr dilution were synergised with those of PRP pre-cooling at 4 °C, thereby significantly improving endothelial cell migration (Fig. 3B). On the other hand, temperature variation failed to modify the reorganisation of endothelial cells in tubular structures, regardless of whether PRPr were diluted or not (Fig. 3C).

The cryoprecipitate obtained after slowly thawing fresh frozen plasma is primarily composed of fibrinogen, von Willebrand Factor (VWF), and fibronectin^10,11^ and it works as a semisolid scaffold for re-epithelisation and angiogenesis during regeneration^11,12^. Thus, the ability of the cryoprecipitate to enhance angiogenesis mediated by PRPr was tested *in vitro*. While HMEC-1 proliferation induced by PRPr was not altered following cryoprecipitate addition (Fig. 3D), cell migration and tubule formation were significantly improved (¥P < 0.05) (Fig. 3E,F). Remarkably, the most important variable to improve HMEC-1 tubule formation was cryoprecipitate supplementation, regardless of the temperature or dilution effect. The combination of the three optimised variables (cold preconditioning, dilution, and supplementation with plasma cryoprecipitate) resulted in a marked improvement in PRPr-mediated angiogenesis. Specifically, comparisons between the non-optimised PRP (PRPr: PRP incubated at 37 °C and PRPr without dilution or supplements) and the optimised PRP (O-PRPr: PRP preincubated at 4 °C, and PRPr diluted to 25% and supplemented with plasma cryoprecipitate) indicated a 2.5-, 2.7-, and 2-fold increase in proliferation, migration and tubule formation, respectively.

### Angiogenesis mediated by PRPr from diabetic patients is improved by optimisation

**Figure 4:**
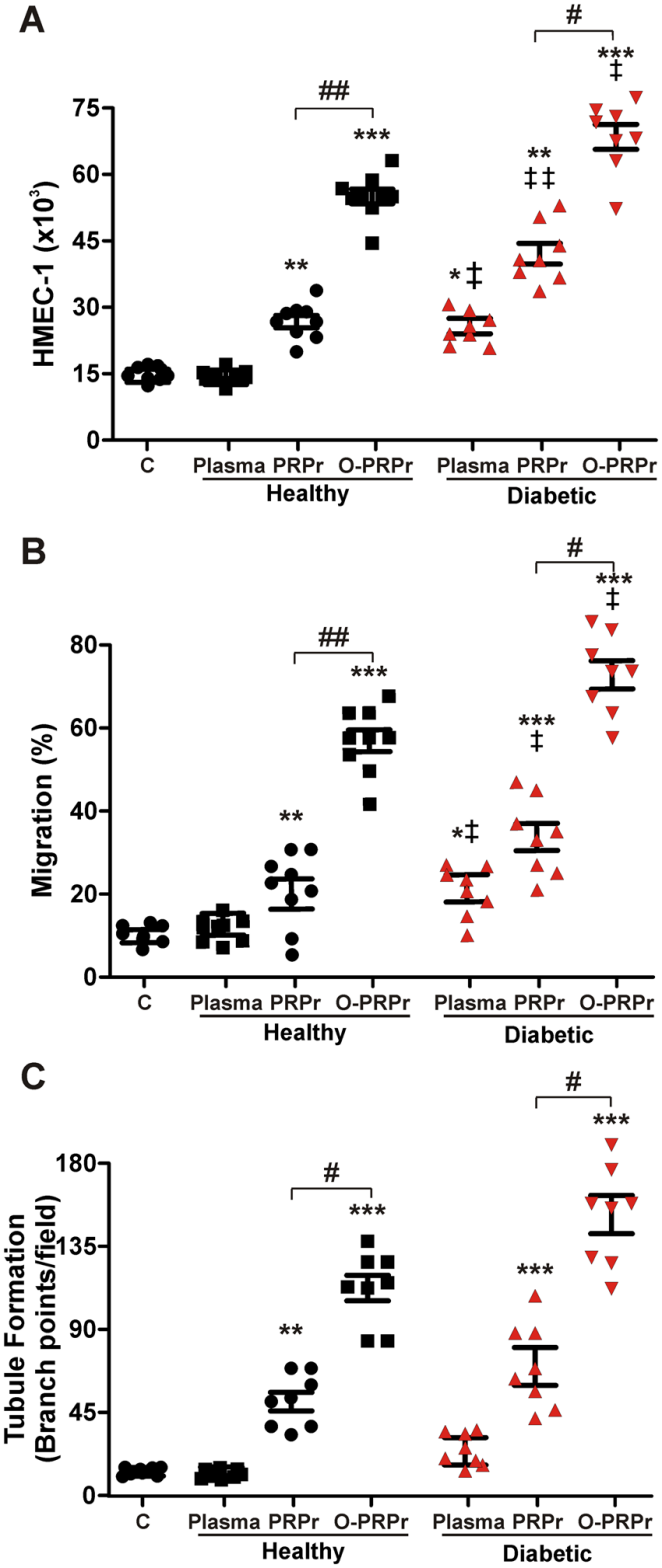
Angiogenesis mediated by PRPr from diabetic patients is increased by optimisation. Platelet-poor-plasma (plasma) and PRP were obtained from blood samples obtained from healthy or diabetic patients. PRPr was prepared as non-optimised (PRPr: PRP pre-incubated at 37 °C; PRPr without dilution or supplements) or optimised PRP (O-PRPr: PRP pre-incubated at 4 °C; PRPr diluted and supplemented with plasma cryoprecipitate). Saline containing 2% FBS was used as the control. (**A**) Endothelial proliferation, (**B**) migration, and (**C**) tubule formation were determined *in vitro* (n = 8 per group; **P < 0.01, ***P < 0.001 vs. control; ^#^P < 0.05, ^##^P < 0.01, ^###^P < 0.001 vs. PRPr; ^‡^P < 0.05, ^‡‡&^P < 0.01 vs. healthy same treatment).

Development of chronic wounds is a common complication in diabetes, and PRP is considered an alternative treatment to enhance wound healing^13,14^. Having established the optimal conditions for the preparation of PRP from healthy donors, we next wondered if this optimisation method would also be able to increase angiogenesis mediated by PRP from type 2 diabetes mellitus (T2DM) patients. With this aim, PRPr and O-PRPr were prepared from T2DM patient blood (the clinical characteristics of patients and healthy donors are shown in Table 1). As shown in Fig. 4A–C, regardless of the preparation method, angiogenic responses, including proliferation and migration, triggered by PRPr from diabetic patients were significantly higher than those induced by PRPr of healthy donors (‡P < 0.05). Furthermore, unlike the platelet-poor plasma from healthy donors, endothelial proliferation and migration (Fig. 4A,B) were induced *per se* by plasma from diabetic patients. Apart from these differences, the three angiogenic responses induced by PRPr from diabetic patients were significantly increased after PRP optimisation, as similarly observed in healthy donors.

**Table 1:** Clinical characteristics of the studied groups.

	Healthy	Diabetic
n	8	8
Age (years)	48 ± 3	52 ± 3
Males (%)	50	50
Systolic blood pressure (mmHg)	123 ± 18	125 ± 7
Diastolic blood pressure (mmHg)	73 ± 10	74 ± 8
Glucose (mg/dL)	84 ± 6	119 ± 28
HbA1c (%)	4.2 ± 0.3	6.2 ± 1.2
HDL-C (mg/dL)	64 ± 3	49 ± 9
TG (mg/dL)	149 ± 14	166 ± 65
White blood cells (x10^9^/L)	5.5 ± 0.4	5.6 ± 0.3
Platelets whole blood (x10^9^/L)	168 ± 13	174 ± 14

### PRP optimisation improves angiogenesis and tissue regeneration *in vivo*

**Figure 5:**
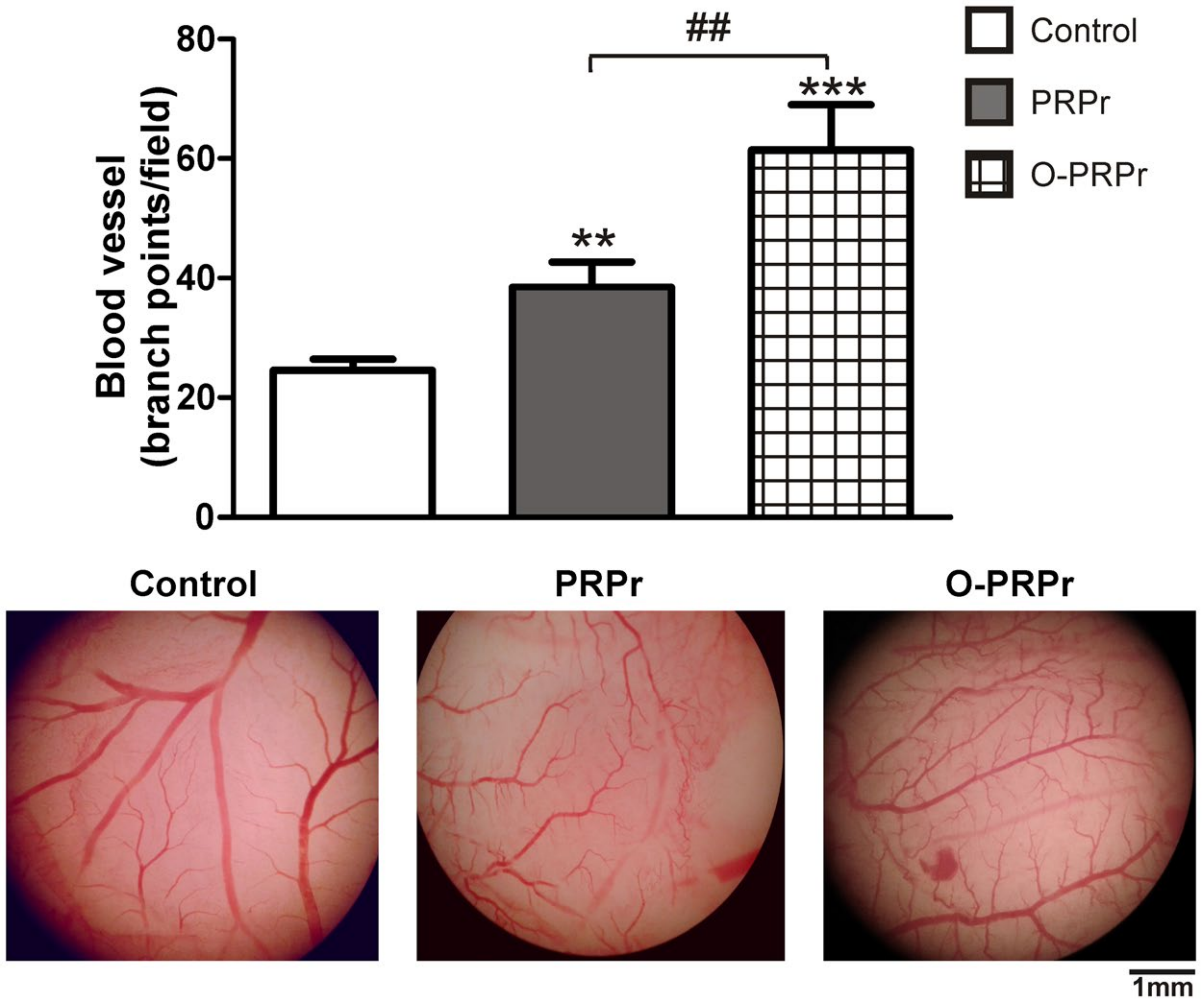
Optimisation of PRP increases angiogenesis *in vivo*. Paper. filters embedded with PRPr or O-PRPr were placed on the CAM. Saline was used as the control. After 24 h, filters were removed and photographed under 2X magnification. The number of blood vessel branch points per field was analysed using the ImageJ software (n = 35–46; **P < 0.01, ***P < 0.001 vs. control; ^##^P < 0.01 vs. PRPr).

To study the relevance of the *in vitro* findings, optimisation of PRP was analysed in a model of angiogenesis *in vivo*. Paper filters embedded with PRPr or O-PRPr were incubated over a chorioallantoic membrane (CAM) of quail embryos for 24 h. Angiogenesis was determined as the number of new thin vessels branching from pre-existing large blood vessels. Sprouting of small blood vessels from pre-existing large vasculature was triggered by PRPr; this phenomenon was significantly (P < 0.01) increased following optimisation (Fig. 5).

**Figure 6:**
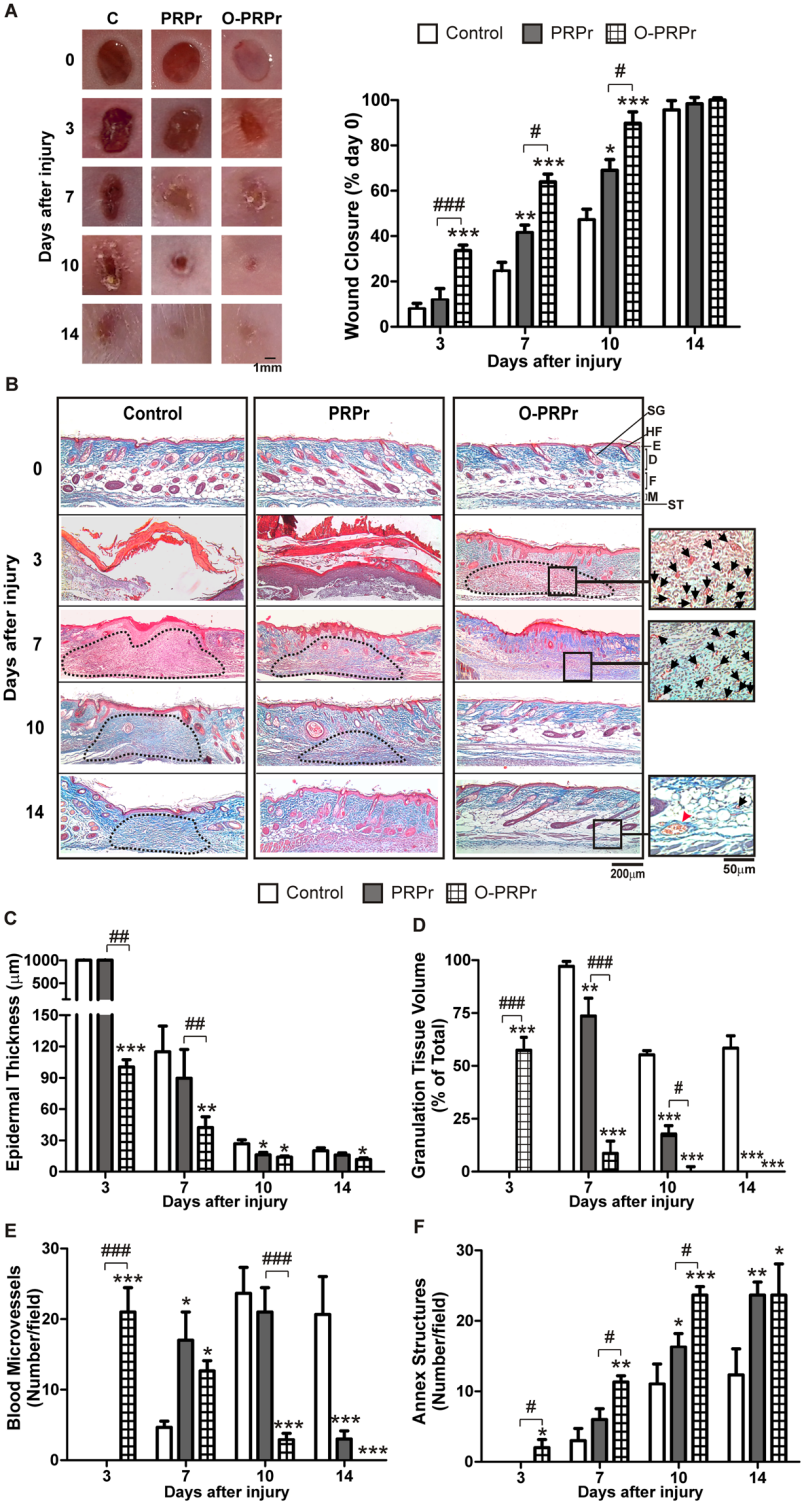
PRP optimisation accelerates mouse skin regeneration. Four round full-thickness excisional wounds, 3 mm in diameter, were generated in the back skin of female BALB/c mice (8–10 weeks old). PRPr or O-PRPr obtained from other mice was injected subcutaneously in the periphery of the wounds. Saline was used as the control. Healing was analysed at 3, 7, 10, and 14 days post-injury. (**A**) Wounds were photographed, and the perimeter of the wound area was determined using the ImageJ software and expressed as a percentage of the area on day 0. (**B**) Skin biopsies were stained with Masson’s trichrome. Images were captured using an inverted microscope. (**C**) Epidermal thickness, (**D**) granulation tissue volume (dotted lines), (**E**) immature blood microvessels containing intraluminal erythrocytes, and (**F**) annex structures (hair follicles and sebaceous glands) were quantified using the ImageJ software. SG, sebaceous gland; HF, hair follicle; E, epidermis; D, dermis; F, fat layer; M, muscle layer; ST, subcutaneous tissue. (Magnification 100× and 200× , n = 4–15; *P < 0.05, **P < 0.01, ***P < 0.001 vs. control; ^#^P < 0.05, ^##^P < 0.01, ^###^P < 0.001 vs. PRPr).

Although the development of new vessels is one of the first and crucial steps during tissue regeneration, this phenomenon also requires the maturation of other tissues^1^. To further investigate this complex process, the ability of PRP optimisation to improve wound healing was analysed using a murine model of skin regeneration. Saline (control), PRPr or O-PRPr were subcutaneously injected in the periphery of four round full-thickness excisional wounds generated in the back skin of animals, and healing was analysed at 3, 7, 10, and 14 days post-injury. After 7 and 10 days, the percentage of wound closure mediated by PRPr was significantly higher than that by the control, achieving complete closure at day 14 (Fig. 6A). Moreover, healing kinetics were accelerated by O-PRPr. Specifically, while only 8–11% wound perimeter closure was observed after 3 days in both control and PRPr, 35 ± 2% closure was attained after 3 days of O-PRPr injection. Furthermore, the increased healing capacity of O-PRPr was observed at day 7, with almost complete wound closure at day 10 (Fig. 6A).

To further understand the regenerative properties of O-PRPr, a histological examination of skin biopsies stained with Masson’s trichrome was performed (Fig. 6B). Four different histological criteria were used to evaluate wound healing: (1) epidermal regeneration quantified as a reduction in hyperplastic neoepidermis thickness (Fig. 6C); (2) granulation tissue development as a provisional matrix for regeneration (Fig. 6D); (3) incipient vascularisation of granulation tissue by small neoformation blood vessels (Figs. 6E); and (4) regeneration of skin mature annex structures, including sebaceous glands (SG) and hair follicles (HF) (Fig. 6F).

Images revealed that, after 3 days of control or PRPr treatment, the neoepidermis remained thick and hyperplastic due to the presence of stratum corneum (red) and a thick papillary dermis (adjacent below stratum corneum) (Fig. 6B and C). In contrast, a significant reduction in epidermal thickness was observed after 3 days of O-PRPr injection (Fig. 6C), together with the early development of granulation tissue (dotted lines Fig. 6B and D) and the formation of immature small vessels containing intraluminal erythrocytes (black arrow Fig. 6B and E).

After 7 days, reduction in epithermal thickness and development of granulation tissue was also observed in control- and PRPr treated-wounds (Fig. 6C,D). Again, the progression of healing after O-PRPr injection was notably accelerated at day 7, with a significant reduction in granulation tissue containing immature blood vessels (Fig. 6D,E). Furthermore, dermal collagen deposition and maturation (indicated by the blue colour in Masson’s trichrome stained tissue) was observed after 7 days of O-PRPr (Fig. 6B). The maturation of connective tissue was accompanied by the incipient regeneration of annex structures (Fig. 6F).

Ten days after injury, normal collagen deposition (blue staining, Fig. 6B) and epidermal thickness (Fig. 6C) were restored in all treatment groups. Granulation tissue containing an increased number of blood microvessels remained elevated in control wounds (Fig. 6D,E), but still allowed for the development of annex structures on the periphery (Fig. 6F). Compared to the controls, a slight but significant reduction in granulation tissue volume (Fig. 6D) and faster development of annex structures (Fig. 6F) was detected after 10 days of PRPr. On the other hand, a complete regeneration of skin structural layers (epidermis, dermis, fat, and muscle), maturation of tissue blood vessels (red arrow) and full restoration of dermal annex structures was observed after 10 days of O-PRPr treatment (Fig. 6B). In contrast, complete reduction of granulation tissue containing immature blood vessels and full restoration of dermal annex structures were evidenced only after 14 days of PRPr treatment (Fig. 6D–F) while at the same time point, incomplete regeneration was observed in control wounds.

### Inhibition of PRP regenerative activity by ASA is partially compensated by optimisation

We and others have demonstrated that angiogenesis mediated by platelets is inhibited by ASA^15–19^. Considering the massive use of ASA as an anti-inflammatory drug (500–1500 mg/day) or as anti-aggregatory therapy for cardiovascular disorders (100 mg/day), it is surprising that, to date, there is no available information about a possible effect of this anti-platelet drug on the PRP regenerative capacity. Thus, we next evaluated the effect of ASA on PRP-mediated angiogenesis. PRP was pre-incubated with high (0.5 mM) or low (0.1 mM) doses of ASA for 30 min, following which PRPr and O-PRPr were generated and used to induce angiogenesis *in vitro* and *in vivo*.

**Figure 7:**
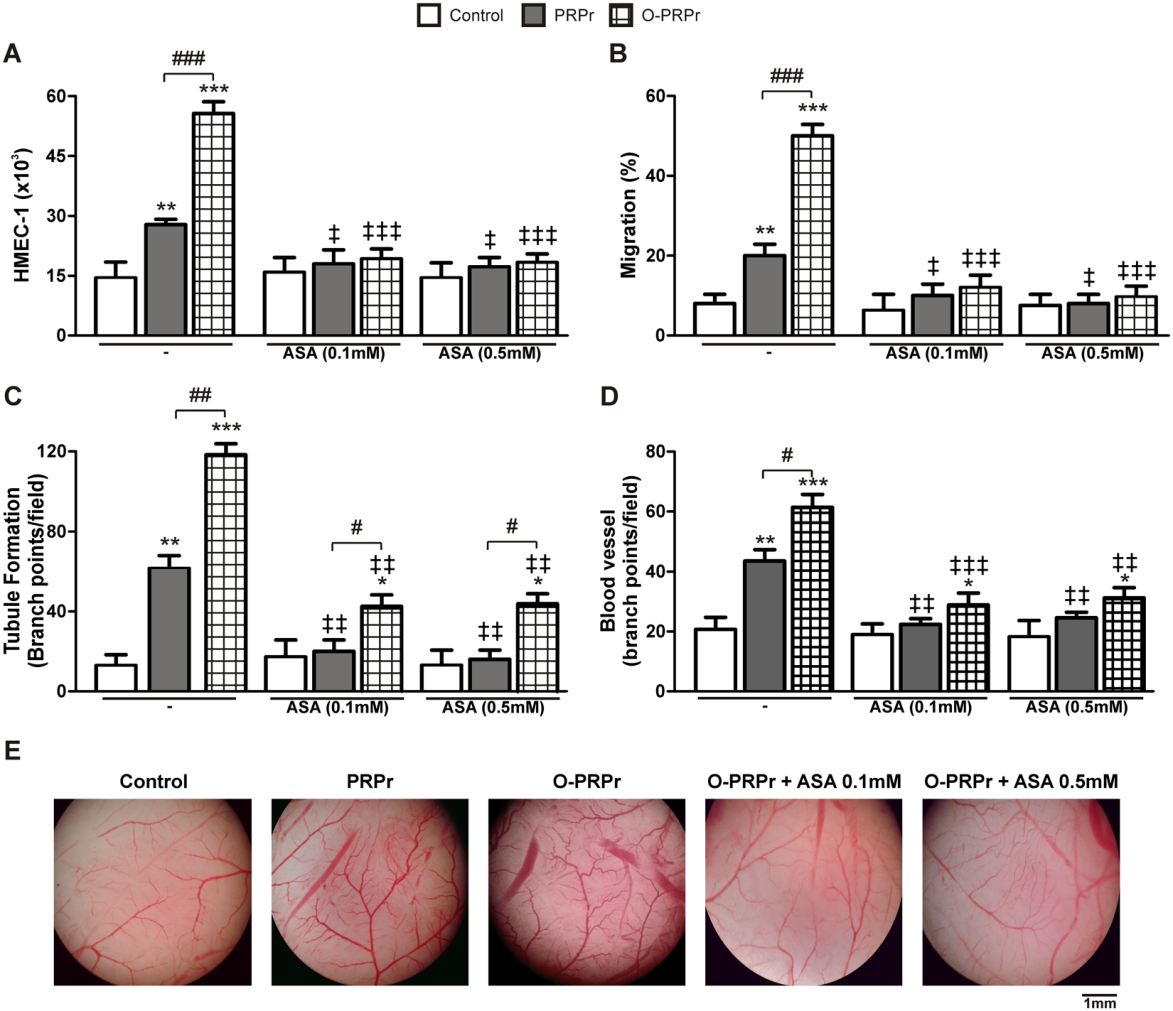
Angiogenesis mediated by PRPr is inhibited by ASA and partially reversed by optimisation. PRP was incubated with ASA (0.1 or 0.5 mM) for 30 min, and then PRPr or O-PRPr was obtained and used to induce angiogenesis *in vitro*: (**A**) Endothelial proliferation, (**B**) migration, (**C**) tubule formation, and *in vivo* (**D**) blood vessel ramification over the CAM. (**E**) Microscopic CAM images represent one independent experiment. (n = 7–25, *P < 0.05, **P < 0.01, ***P < 0.001 vs. control; ^#^P < 0.05, ^##^P < 0.01, ^###^P < 0.001 vs. PRPr; ^‡^P < 0.05, ^‡‡^P < 0.01, ^‡‡‡^P < 0.001 vs. the same treatment without ASA).

As shown in Fig. 7, all *in vitro* and *in vivo* angiogenic responses mediated by PRPr were completely dampened by pre-incubation with both low and high concentrations of ASA. Although it could be argued that the inhibition of angiogenesis was by the direct action of ASA on endothelial cells, this hypothesis was ruled out because the addition of PRPr that had been supplemented with 0.5 and 0.1 mM ASA after PRP coagulation failed to modify any of the *in vitro* angiogenic responses (Suppl. Fig. 2), indicating that the inhibitory effect was exerted on platelets and not endothelial cells. However, higher ASA concentrations were required to inhibit angiogenesis (Suppl. Fig. 2). Remarkably, the inhibitory effect of ASA on tubule formation *in vitro* and blood vessel development *in vivo* was partially reversed after the optimisation of PRPr (Fig. 7C–E).

**Figure 8:**
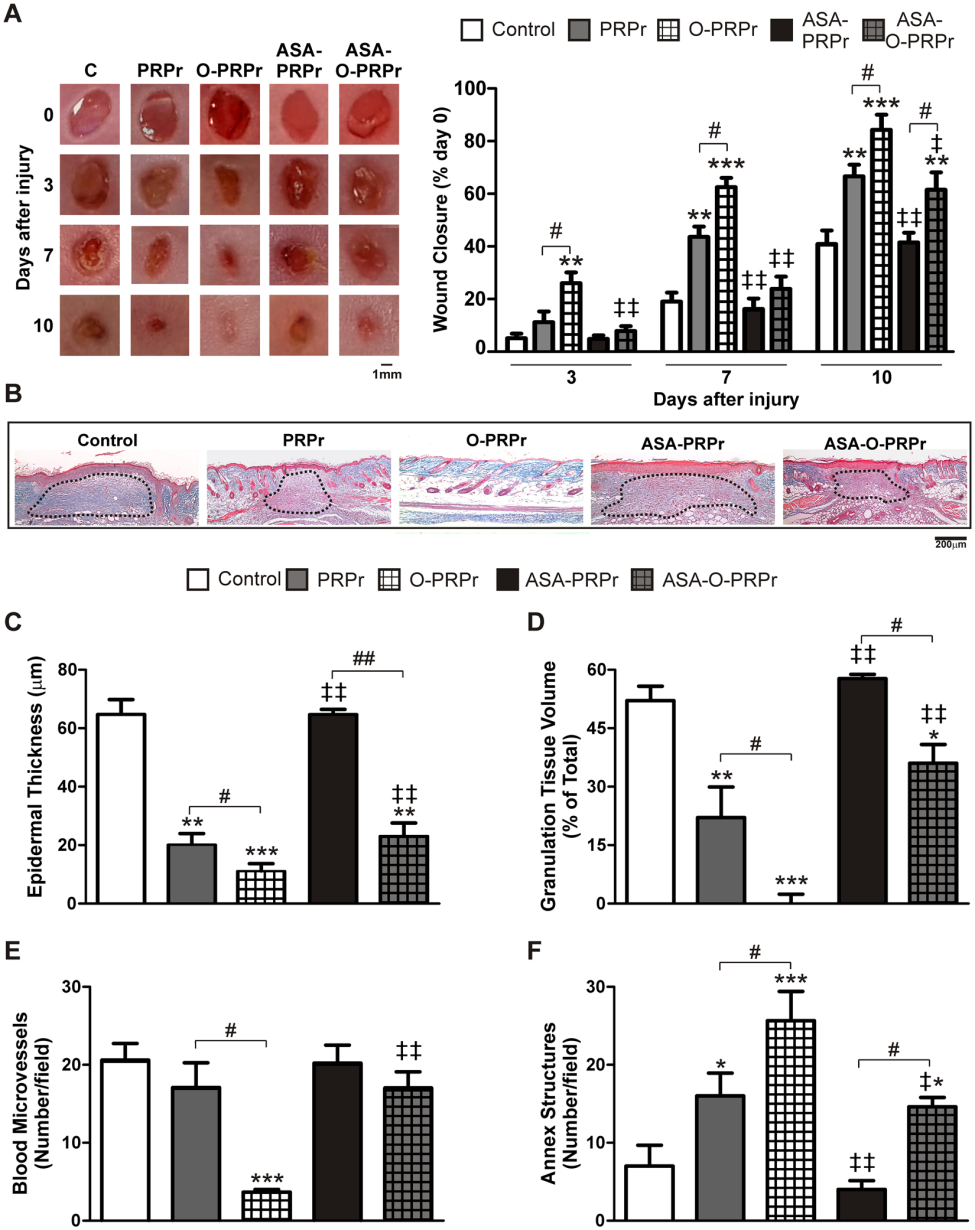
Inhibition of PRP-regenerative activity by ASA is partially compensated for by optimisation. Four round full-thickness excisional wounds, 3 mm in diameter, were generated in the back skin of female BALB/c mice (8–10 weeks old). PRPr or O-PRPr obtained from ASA- or non-ASA- treated mice was injected into the wounds generated in ASA-treated mice. Injection with saline was used as the control. The healing process was analysed at 3, 7, and 10 days post-injury. (**A**) Wounds were photographed, and the perimeter of the wound area was determined using the ImageJ software and expressed as a percentage of the area on day 0. (**B**) Skin biopsies were obtained at day 10 and stained with Masson’s trichrome. Images were captured using an inverted microscope. (**C**) Epidermal thickness, (**D**) granulation tissue volume (dotted lines), (**E**) immature blood microvessels containing intraluminal erythrocytes, and (**F**) annex structures (hair follicles and sebaceous glands) were quantified using the ImageJ software. (Magnification 100X, n = 7; *P < 0.05, **P < 0.01, ***P < 0.001 vs. control; ^#^P < 0.05, ^##^P < 0.01 vs. PRPr; ^‡^P < 0.05, ^‡‡^P < 0.01 vs. the same treatment without ASA).

Finally, to mimic the potential use of PRP in cardiovascular patients who take ASA daily, we analysed the *in vivo* regenerative ability of PRPr or O-PRPr in mice treated with a low dose of ASA. Injection of PRPr and O-PRPr (obtained from non-ASA treated mice) in ASA-treated animals showed improved wound closure (Fig. 8A) similar to that observed in non-ASA treated animals (Fig. 6A), indicating that ASA intake does not interfere with the local haemostatic effect of exogenous PRPr. Moreover, as in the *in vitro* studies, we observed that compared to PRPr from non-ASA-treated mice, injection of PRPr obtained from ASA-treated mice (ASA-PRPr) failed to induce wound healing, and the addition of ASA-O-PRPr partially reversed the inhibitory effect of ASA (Fig. 8). Specifically, a significant improvement in wound closure was observed after 10 days of treatment with ASA-O-PRPr but not ASA-PRPr (Fig. 8A). Histological analysis of biopsies revealed the notable presence of a hypertrophic neoepidermis, granulation tissue, and immature blood vessels in ASA-PRPr-treated wounds (Fig. 8C–E). In contrast, reduction in epidermal thickness and granulation tissue as well as regeneration of dermal annex structures was significantly promoted by ASA-O-PRPr compared to that induced by ASA-PRPr (Fig. 8C–F).

## Discussion

Although PRP is used as a source of platelet-derived growth factors in regenerative medicine, its effectiveness remains controversial, partially due to the lack of large controlled clinical trials and the absence of PRP preparation protocols based on the regenerative role of platelets. Herein, we aimed to optimise current PRP preparation protocols by evaluating angiogenic and regenerative responses induced by PRP. Our results indicate that preincubation of PRP at 4 °C, PRPr dilution, and plasma cryoprecipitate supplementation improve the angiogenic and regenerative properties of PRP compared to those of the PRP obtained by current methods.

We observed that both WPr and diluted PRPr were more efficient than PRPr as inducers of *in vitro* angiogenic responses, such us endothelial proliferation, migration, and tubule formation. These results are in agreement with our previous findings demonstrating that angiogenic responses induced by releasates derived from PAR-1- or PAR-4-stimulated WP were higher than those mediated by PRP^7^. In addition, it was demonstrated that, while the proliferation of osteoblasts^20^ or Schwann cells^21^ is maximally induced by a basal medium supplemented with 20% PRPr, cell growth is inhibited at higher PRPr concentrations (>40%). It is known that compared to platelets, plasma contains higher levels of anti-angiogenic molecules^7,8^, including matrix-derived inhibitors (e.g., endostatin, thrombospondin, and tumstatin) as well as non-matrix derived inhibitors (e.g., angiostatin, the soluble version of VEGFR-1 (sFlt-1), pigment epithelium-derived factor (PEDF), and prolactin) which may competitively and reversibly interfere with several growth factor receptors^22^. Therefore, it is conceivable that the removal of plasma accounts for the higher angiogenic activity of WP compared to that of PRP. Interestingly, we observed that, while PRPr dilution to 25% improved its ability to induce HMEC-1 growth, higher dilutions decreased the effect until it was completely lost at 6% PRPr. These data suggest that the optimal angiogenic activity achieved by PRPr dilution is the result of a balance between reduction of anti-angiogenic plasma molecules and maintenance of the minimal amount of platelet-derived growth factors required to induce angiogenesis. Indeed, it has been reported that the high-affinity interaction between angiogenic molecules and their receptors requires a low concentration of ligands^22^.

Another strategy to improve angiogenesis mediated by PRP is to increase the amount of growth factors released from platelets. PRP is usually processed at 37 °C or RT (20–25 °C); however, it is traditionally known that lower temperatures prime platelet activation and trigger the release of alpha granules^9^. Accordingly, we observed that the release of VEGF, EGF, bFGF, IL-17, and IL-8, but not PDGF, was significantly increased when PRP was incubated at 4 °C before inducing coagulation. The intriguing observation that the release of PDGF was not modified by temperature may be explained by the new theories of platelet granule secretion which recognise the existence of alpha granule subtypes associated with variable morphologies, protein cargos, and release mechanisms^23–25^. Remarkably, suppressing the effects of molecules, such as EGF or VEGF, was not enough to reverse the enhanced angiogenesis induced by 4 °C preconditioning. In agreement with our previous reports^15,26^, these results indicate that angiogenesis mediated by platelets is a complex process, not associated with a particular growth factor, but with the combined activity of several proangiogenic molecules. Our data, demonstrating that phosphorylation of p38–an important signalling involved in granule secretion from platelets–was increased when platelets were exposed to 4 °C and that a complete reversion of cold preconditioning enhanced angiogenesis was observed in the presence of the specific inhibitor of p38 phosphorylation, indicated that cold preconditioning primes platelet-mediated angiogenesis by enhancing activation of p38.

Additionally, in agreement with previous findings regarding the effect of low temperature on coagulation^27,28^, we observed that the viscosity of PRP at 37 °C was quickly altered ~1 min after CaCl_2_ addition, a phenomenon associated with the initiation of coagulation. In contrast, when PRP was pre-incubated at 4 °C for 30 min and then incubated with calcium at 37 °C, the initiation of coagulation was markedly delayed by up to ~6 min (Suppl. Fig. 3). These data suggest that apart from increasing angiogenesis, cold pre-incubation may also be beneficial for delaying PRP coagulation inside the syringe in protocols in which PRP is administered through injection immediately after the addition of calcium (e.g. intraarticular, intraosseous, or subcutaneous)^29^, thereby reducing pain associated with the injection of viscous solutions^30,31^. On the other hand, several controversies are associated with storage of platelets at 4 °C, since it may induce platelet activation as well as their rapid clearance from circulation^32^. However, considering that the application of PRP for tissue regeneration is local and not systemic, these concerns regarding the safety and quality control of refrigerated platelets may be unimportant in the clinical setting of regenerative medicine.

Supplementation with the cryoprecipitate obtained after slowly thawing fresh frozen plasma was also tested as an approach to enhance the pro-angiogenic effect of platelets. This biomaterial is used in regenerative treatments as a provisional matrix that offers a semi-solid scaffold for re-epithelisation and angiogenesis, two critical processes during tissue regeneration^11,12^. Our findings demonstrate that endothelial migration and tubule formation mediated by PRPr were enhanced following cryoprecipitate supplementation. This effect could be associated with the fact that the cryoprecipitate is rich in fibrinogen, fibronectin, and VWF, three adhesion molecules that bind to endothelial integrins and provide a scaffolding surface necessary for cell adhesion, migration, and differentiation^10,11^. In contrast to tubule formation and migration, the proliferation of endothelial cells was not modified by cryoprecipitate supplementation. The lack of mitogenic activity of the cryoprecipitate is not surprising, considering that intracellular signalling pathways governing migration and tubule formation, although possibly overlapping, are distinctly separated from those regulating proliferation^33^.

PRP is currently administered not only to healthy individuals (to improve tissue recovery after surgery, or muscle and bone repair after trauma) but also for healing injuries associated with inflammatory diseases (burns, diabetic foot ulcers, and rheumatoid arthritis)^5^. Intriguingly, the regenerative capacities of PRP derived from patients with inflammatory diseases remain uncertain. We observed that, like PRPr from healthy donors, angiogenesis triggered by PRPr from patients with T2DM–a metabolic disease with a component of low-grade chronic inflammation–was significantly improved following optimisation, suggesting that these protocols may also be considered for improving autologous treatment of chronic wounds in diabetic patients. Furthermore, under our experimental conditions, the angiogenic responses induced by PRPr from diabetic patients were greater than those triggered by PRPr from healthy donors. This phenomenon was not surprising, considering the high levels of growth factors constitutively circulating in the plasma of diabetic patients including EGF, FGF-2, G-CGF, TNFα, and VEGF^34,35^. Controversially, a recent study performed by Miao *et al*. demonstrated that releasates from PAR-1- or PAR-4-activated platelets from healthy or diabetic patients exert similar levels of endothelial tubular formation *in vitro*^36^. Differences between the results presented by Miao and those of the current study may be associated with the fact that, while they used WP, we used PRP, adding a possible effect of plasma. Apart from the experimental differences, both studies suggest that the ability of platelets and/or PRP to modulate angiogenesis is preserved in diabetic patients, emphasising that the primary cause of diabetic chronic wounds is associated with severe neuropathy and vascular dysfunction^5,13,14,34^ and not necessarily with impaired physiologic mechanisms involved in wound healing, such as angiogenesis.

Beyond angiogenesis, tissue regeneration also requires the maturation of other tissues as well as fibroblast migration and activation, extracellular cell matrix reorganisation, and cell-cell interactions^1^. Using a murine model of full-thickness skin wound healing, our findings demonstrate that similar to previous reports^37,38^, 7–14 days were required to induce wound closure and skin regeneration after traditional PRPr treatment. Remarkably, this phenomenon was accelerated to 3–10 days by treatment with O-PRPr. Unlike mice, full-excisional wound regeneration in humans is a more gradual and complex process that requires between months and years, depending on the wound characteristics^1^. Bearing this in mind, the acceleration of wound healing by PRP optimisation in patients could represent a reduction in hospitalisation costs, a decrease in analgesic and anti-inflammatory indications, and may lower risks of non-healing complications including infection, lost limb functionality, and amputation^39,40^, that are commonly associated with impaired skin healing and regeneration in diabetic chronic wounds and dermal burns, which are target diseases for PRP treatment.

To date, there is no available information on the possible effects of anti-platelet drugs on the regenerative capacity of PRP. In this context, we studied the effect of ASA, a common anti-platelet drug used as therapy for several anti-inflammatory diseases and for the prevention of cardiovascular events. In agreement with previous reports,^15–19^ our findings demonstrate that angiogenesis mediated by PRPr was completely abolished by the pre-treatment of PRP with ASA. This phenomenon was associated with inhibition of the cyclooxygenase-1 signalling pathway, which is crucial for granule secretion and the consequent release of platelet-derived growth factors, such as VEGF, EGF, bFGF, IL-17, and IL-8 (Suppl. Fig. 4).

Beyond angiogenesis, mouse skin wound healing was also completely blocked by PRPr derived from ASA-treated mice, demonstrating for the first time that this anti-platelet drug negatively affects tissue regeneration mediated by PRP. Remarkably, and in contrast with traditional PRP, the inhibitory effect of ASA was partially reversed following PRP optimisation. Moreover, angiogenic responses including tubule formation (*in vitro*) and blood vessel development (*in vivo*), as well as mouse skin regeneration, were significantly induced after the infusion of ASA-O-PRPr.

In conclusion, our findings demonstrate that compared to non-optimised PRP (traditional method at 37 °C, without dilution or supplements), a combination of the three PRP optimising variables (PRP preincubation at 4 °C, PRPr diluted to 25% and supplemented with plasma cryoprecipitate) exerted an additive effect, resulting in a marked improvement in PRPr-mediated angiogenesis. These simple and economic variables induce more efficient tissue repair than that induced by PRP obtained by the current preparation methods for regenerative medicine, not only using PRP from healthy donors but also PRP obtained from diabetic patients. Moreover, the inhibitory effect that ASA exerts on regeneration mediated by PRP was reversed by optimisation, suggesting that this method may be useful for improving tissue regeneration in patients treated with ASA.

A controlled randomised clinical trial is warranted to confirm the usefulness of this protocol in the clinical setting.

## Methods

### Ethics statements

Human blood studies were conducted according to the principles expressed in the Declaration of Helsinki and after receiving the approval of the Ethic Committee of the National Academy of Medicine (ID 79-1006-2015). All subjects provided informed written consent for the collection of samples and subsequent analysis.

Animal studies received the approval of the Institutional Committee for Care and Use of Laboratory Animals (CICUAL, ID 0010-2016).

### Human blood samples

Blood samples were obtained from healthy donors who had not taken non-steroidal anti-inflammatory drugs during the 10 days before sampling, or from T2DM patients diagnosed according to the American Diabetes Association criteria^41^. Patients with active infections or under chronic treatment with ASA were excluded. Patients received standard care by their treating physicians and were treated with metformin and statins at different doses. A routine examination was performed. Systolic and diastolic blood pressures were determined using a standardised protocol. Venous blood samples (20 mL) were obtained after a 12-h overnight fast. Fasting plasma glucose was determined by the glucose-oxidase method (GLU Glucose GOD-PAP, Roche Diagnostics, Mannheim, Germany) in a Hitachi 727 auto-analyser. Levels of triglycerides (TG) and high-density lipoprotein cholesterol (HDL-C) were determined in the serum by standard enzymatic methods using commercial kits (TG Triglycerides GPO-PAP and Phosphotungstate Precipitant, Roche Diagnostics, Mannheim, Germany) with a Hitachi 727 auto-analyser. HbA1c was measured by HPLC using a commercial kit (Roche Diagnostics, Mannheim, Germany). As a control group, eight healthy individuals of the same age and gender were also evaluated.

### Preparation of PRPr

PRPr preparation was adapted from previously described methods^21^. Briefly, PRP was obtained from anticoagulated human blood (sodium citrate 3.8%) by centrifugation at 180 × *g* for 10 min. PRP was coagulated by adding CaCl_2_ (22 mM) and clots were allowed to retract for 40 min at 37 °C. Following clot removal, the exudate was centrifuged at 890 × *g* for 10 min and the supernatant containing the platelet-derived growth factors (PRPr) was stored at −80 °C until further use in assays to analyse growth factor concentration or angiogenesis.

### Preparation of WPr

WPr were obtained as previously described^15^. PRP was centrifuged in the presence of PGI_2_ (75 nM) at 890 × *g* for 10 min, washed in washing buffer (pH 6.5) at 890 × g for 10 min, and resuspended in the original volume of Tyrode’s buffer. Activation was induced by adding human alpha-thrombin (1 U/mL, Enzyme Research Laboratories) and CaCl_2_ (22 mM) for 40 min at 37 °C. Cells then were centrifuged and releasates were stored at −80 °C until use in the assays performed.

In some experiments, platelet pellets with similar cell concentrations were resuspended in Tyrode’s buffer supplemented with different concentrations of plasma. Following the addition of thrombin and CaCl_2_, clots were removed, samples were centrifuged and releasates were stored at −80 °C.

### Plasma cryoprecipitate isolation

Plasma cryoprecipitate preparation was adapted from previously described methods^42^. Briefly, 40 mL of anticoagulated human blood (sodium citrate 3.8%) was centrifuged at 600 × *g* for 20 min. Platelet-poor-plasma was collected and frozen at −80 °C for 2 h and then thawed at 4 °C for 24 h. Following centrifugation at 1600 × *g* 15 min, plasma was discarded, and the pellet was resuspended in 4 mL of saline solution.

### Optimisation of PRP

To determine the effect of temperature preconditioning, PRP was incubated at 37 °C, 23 °C, or 4 °C for 30 min. Next, CaCl_2_ (22 mM) was added and clotting was induced for 40 min at 37 °C. Clots were removed and PRP supernatants were obtained following centrifugation. PRPr was serially diluted with saline or supplemented with 10% cryoprecipitate.

In some experiments PRP was incubated with ASA (0.1 or 0.5 mM) or p38 MAP kinase inhibitor (SB203580, 10 µM, InvivoGen, San Diego, CA, USA) for 30 min, and then at 37 °C or 4 °C for another 30 min. Following coagulation with CaCl_2_, PRPr was diluted or supplemented with cryoprecipitate.

### *In vitro* angiogenesis assays

Human microvascular endothelial cells (HMEC-1) (ATCC cell line) were grown in RPMI supplemented with foetal bovine serum (FBS) (10%), L-glutamine (10 mM) and streptomycin (100 µg/mL), penicillin (100 U/mL), EGF (10 ng/mL), and hydrocortisone (1 µg/mL) at 37 °C in a humidified 5% CO_2_ incubator. Growth factor-enriched medium was totally replaced by either PRPr, WPr (alone or containing different proportions of plasma) or optimised PRPr. In selected experiments, VEGF and EGF receptors were blocked by the addition of anti-human VEGFR2/KDR/Flk-1 (10 µg/ml, R&D Systems, Minneapolis, MN, USA), anti-human-EGFR (40 µg/ml, Cell Signaling, Danvers, MA, USA) or irrelevant IgG controls and incubation for 30 min. Next, endothelial proliferation was induced by addition of recombinant VEGF (20 ng/ml), EGF (20 ng/ml) or with PRPr. The proliferation of endothelial cells (15 × 10^3^) was determined at 24 h by measuring acid phosphatase activity. Migration into scratched monolayers of endothelial cells was analysed at 6 h with ImageJ software and the percentage of migration was calculated as [(wound area at 0 h - wound area at 6 h)/wound area at 0 h]*100. Capillary tube formation in growth factor-reduced Matrigel-coated plates (BD Biosciences) was examined at 18 h under an inverted light microscope and the number of branch points per field was determined with ImageJ software. Endothelial cell viability was determined by labelling cells with a mixture of fluorescent DNA binding dyes (acridine orange and ethidium bromide), as previously described^15^.

### Immunoblotting

Platelets were lysed in loading buffer in the presence of a protease inhibitor cocktail. Equivalent amounts of proteins were subjected to 12% SDS-PAGE and electrotransferred to nitrocellulose membranes (GE Healthcare, Little Chalfont, UK). After blocking, the membranes were incubated overnight at 4 °C with primary antibody pp38-Thr180/Tyr182 (Cell Signaling, Danvers, MA, USA) followed by incubation with an HRP-linked secondary antibody (Santa Cruz Biotechnology, Dallas, TX, USA) for 1 h at 22 °C. Protein bands were visualized using the ECL reaction. Total p38 levels were used for normalization of protein loads and the relative IOD values were calculated using the GEL-PRO software.

### Angiogenesis *in vivo* (CAM assay)

Angiogenesis mediated by human PRPr was studied *in vivo* through the CAM assay. Fertilised quail eggs were incubated at 37 °C for 72 h. Embryos were transferred to a Petri dish and incubated *ex ovo* at 37 °C for 5 days. The emerging vasculature of embryos was incubated with paper filters (Whatman 589/1, 50 mm in diameter, GE Healthcare Life Sciences) embedded with PRPr (two treatments per embryo). After 24 h, filters were removed and photographed under 2X magnification. The number of blood vessel branch points per field was analysed using the ImageJ software^43^.

### Growth factor and cytokine measurement

Levels of angiogenic factors in platelet releasates were determined by ELISA for VEGF (RayBiotech, Inc. Norcross, GA, USA), IL-17, IL-8, bFGF (Biolegend, San Diego, CA, USA), EGF (Life Technologies, Carlsbad, CA, USA), and PDGF (Abcam, Cambridge, UK). The total intra-platelet content of each protein was determined in platelet lysates obtained as previously described^26^.

### Mice

Female BALB/c mice, 8–10 weeks old, originally purchased from Charles River Research Animal Diagnostic Services (Wilmington, MA, USA), were housed in a controlled environment with free access to water and a standard diet in the animal facility of the Institute of Experimental Medicine. Experiments were conducted according to principles set forth in the *Guide for the Care and Use of Laboratory Animals* (8th Ed. NRC, USA) and by the European Parliament and Council concerning the protection of animals used for scientific purposes (Directive 2010/63/EU).

### Model of wound healing in mice

PRP was obtained from orbital blood of mice and incubated at 37 °C or 4 °C for 30 min. PRPr or O-PRPr were prepared using methods similar to those for human samples and were subcutaneously injected in the periphery of four round full-thickness excisional wounds of 3 mm diameter generated in the back skin of anaesthetised mice under aseptic conditions, as previously described^44^. The healing process was analysed at 3, 7, 10, and 14 days post-injury. The back skin of mice was photographed, and the perimeter of the wound area was calculated using the ImageJ software. The results are expressed as a percentage of the area at day 0 when wounds were excised. Mice were culled, and paraffin sections of skin wounds were stained with Masson’s trichrome. Images were captured using an inverted microscope and analysed using the ImageJ software.

In some experiments, PRPr was obtained from ASA-treated animals. ASA was administered for 2 days through oral gavage (17.2 mg/kg, equivalent to 100 mg/day human dosage for the prevention of cardiovascular events)^45^. Next, PRPr or ASA-PRPr were subcutaneously injected into wounds of mice that were given ASA daily during the entire experiment. Macroscopic wound closure was analysed until day 10, when mice were culled and skin biopsies were obtained. The experimental design is illustrated in Suppl. Fig. 5.

### Statistical analysis

Results were expressed as the mean ± SEM. Statistical differences between mean values were determined by one- or two-way ANOVA followed by the Newman-Keuls multiple comparison test using GraphPad Prism 5.0 software. P < 0.05 values were considered as significant.

## Electronic supplementary material

Supplemental Figures
