# Supplementary material for: An optimised protocol for platelet-rich plasma preparation to improve its angiogenic and regenerative properties
Source: Sci Rep. 2018 Jan 24;8:1513. doi: 10.1038/s41598-018-19419-6 (PMC5784112; doi:10.1038/s41598-018-19419-6)
Supplement: Supplementary file 1 — Supplemental Figures [file 41598_2018_19419_MOESM1_ESM.pdf]

SUPPLEMENTAL INFORMATION

An optimised protocol for platelet-rich plasma preparation to improve its angiogenic and regenerative properties

Julia Etulain[1]*, Hebe A. Mena[1], Roberto P. Meiss[2], Gustavo Frechtel[3], Susana Gutt[4], Soledad Negrotto[1], Mirta Schattner[1]

[1]Laboratory of Experimental Thrombosis, Institute of Experimental Medicine, CONICET-National Academy of Medicine. Buenos Aires, Argentina.

[2]Division Experimental Pathology. National Academy of Medicine. Buenos Aires, Argentina.

[3]Genetics and Molecular Biology, Department of Microbiology, Immunology and Biotechnology, School of Pharmacy and Biochemistry, University of Buenos Aires (UBA), Buenos Aires, Argentina.

[4]Nutrition Service, Hospital Italiano, Buenos Aires, Argentina.

***Corresponding Author:**

Julia Etulain, PhD, juliaetulain@hotmail.com; jetulain@hematologia.anm.edu.ar.

Laboratory of Experimental Thrombosis, Institute of Experimental Medicine, CONICET-National Academy of Medicine, Pacheco de Melo 3081, 1425, Buenos Aires, Argentina.

Phone (+54-11)-4805-5759 ext 243

Fax (+54-11)-4805-0712

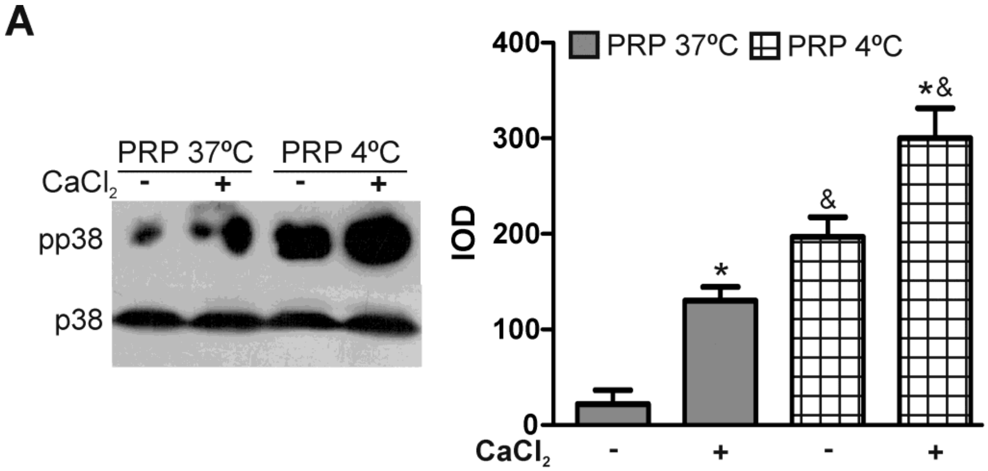

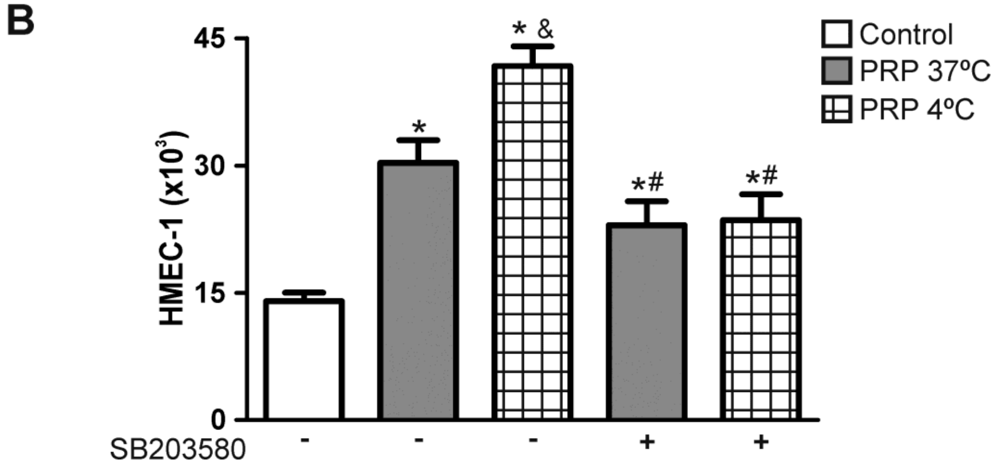

Supplemental Figure 1. Roles of p38 phosphorylation on angiogenesis mediated by cold preincubated PRP. A) PRP was incubated at 37ºC or 4ºC for 30 min, clotted or not with $CaCl_2$, and lysates were immunoblotted with anti-pp38 antibody. Membranes were reprobed with anti-p38 antibody to calculate the relative IOD using GEL-PRO software. B) PRP was preincubated with the inhibitor of p38 (SB203580, 10 µM) 30 min. Then, PRP was exposed to 37ºC or 4°C for 30 min and then clotted by addition of $CaCl_2$. PRPr were used to induce proliferation of HMEC-1 ($15x10^3$). A direct effect of p38 inhibition on endothelial cells was ruled out because the addition of PRPr that had been supplemented with SB203580 after PRP coagulation failed to modify the endothelial proliferation (not shown) (n=3-4, *P<0.05 vs. unstimulated; &P<0.05 vs. 37°C; #P<0.05 vs. without inhibitor).

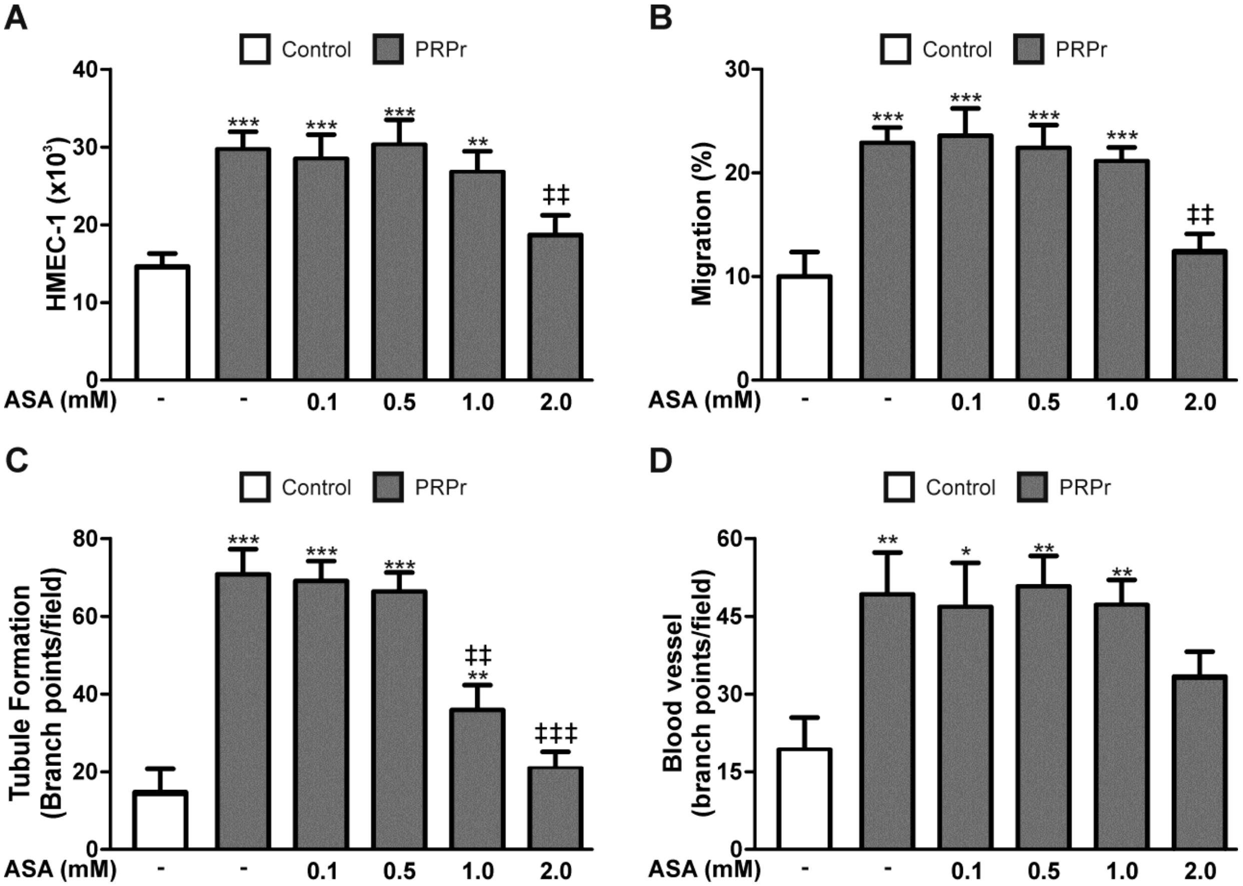

Supplemental Figure 2. Effect of ASA on angiogenesis. PRP was coagulated with $CaCl_2$ for 40 min. After clot removal, PRP releasates (PRPr) were supplemented with ASA (0-2 mM) and used to induce angiogenic responses. Saline plus FBS 2% was used as control. Angiogenesis was determined *in vitro*: A) endothelial proliferation; B) migration; C) tubule formation; and *in vivo* D) blood vessel ramification over CAM. (n=5, *P<0.05, **P<0.01, ***P<0.001 vs. control; ‡‡P<0.01, ‡‡‡P<0.01 vs. PRPr without ASA).

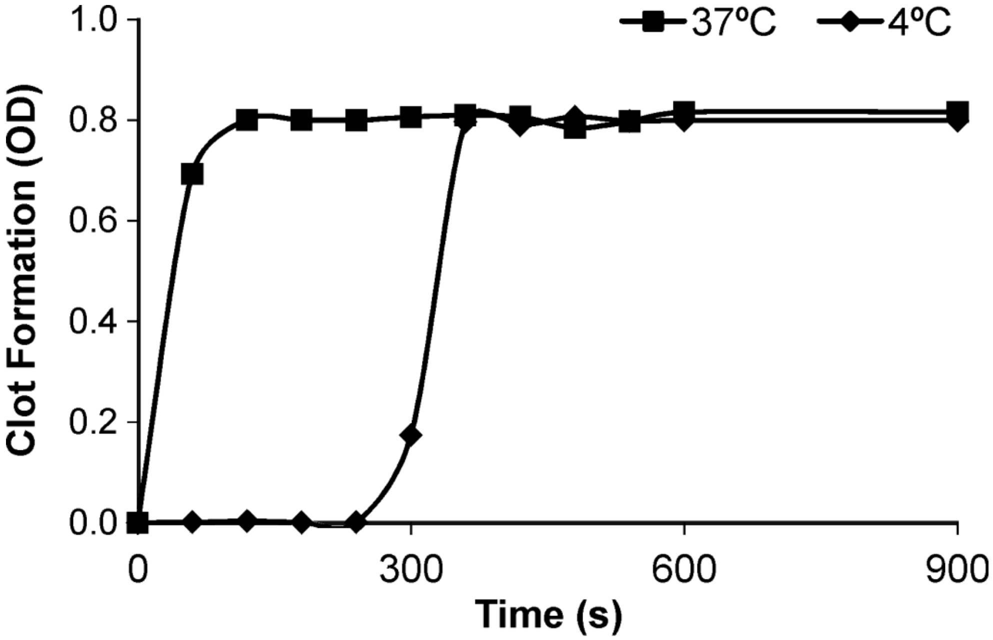

Supplemental Figure 3. Clot formation is delayed after cold preconditioning of PRP. PRP was incubated at 37ºC or 4ºC for 30 min and then placed in 96-well plates. Clotting was initiated by addition of $CaCl_2$ (22mM) and fibrin(clot) formation was monitored by measuring the optical density (405nm) of the plasma on a microplate reader as previous described[1]. The curves are representative of 4 independent experiments.

1 Berckmans, R. J., Sturk, A., van Tienen, L. M., Schaap, M. C. & Nieuwland, R. Cell-derived vesicles exposing coagulant tissue factor in saliva. *Blood* **117**, 3172-3180 (2011).

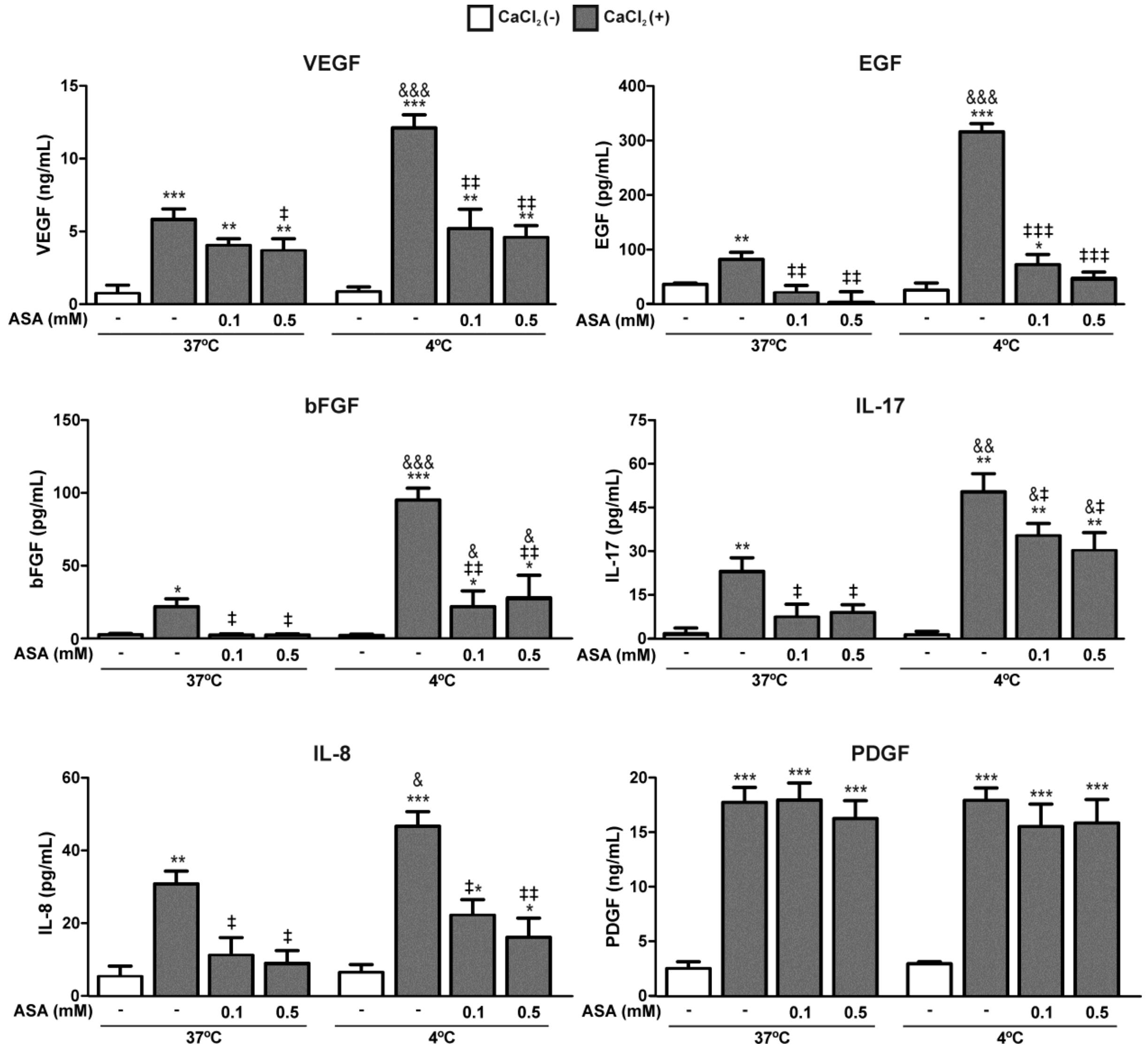

Supplemental Figure 4. Release of platelet-derived growth factors and cytokines is inhibited by ASA. PRP was incubated with ASA (0.1 or 0.5 mM) for 30 min, and then at 37ºC or 4ºC for further 30 min. Coagulation was activated or not with $CaCl_2$ (22 mM) for 40 min. After clot removal and centrifugation, PRP releasates ($CaCl_2$ +) or supernatant of non-coagulated PRP ($CaCl_2$ -) were obtained and the levels of VEGF, EGF, bFGF, IL-17, IL-8, and PDGF were determined by ELISA. (n=4, *P<0.05, **P<0.01, ***P<0.001 vs unstimulated; &P<0.05, &&P<0.01, &&&P<0.001 vs. 37ºC; ‡P<0.05, ‡‡P<0.01, ‡‡‡P<0.001 vs. without ASA). VEGF: vascular endothelial growth factor; EGF: epidermal growth factor; bFGF: basic fibroblast growth factor; PDGF: platelet derived growth factor; IL: interleukin.

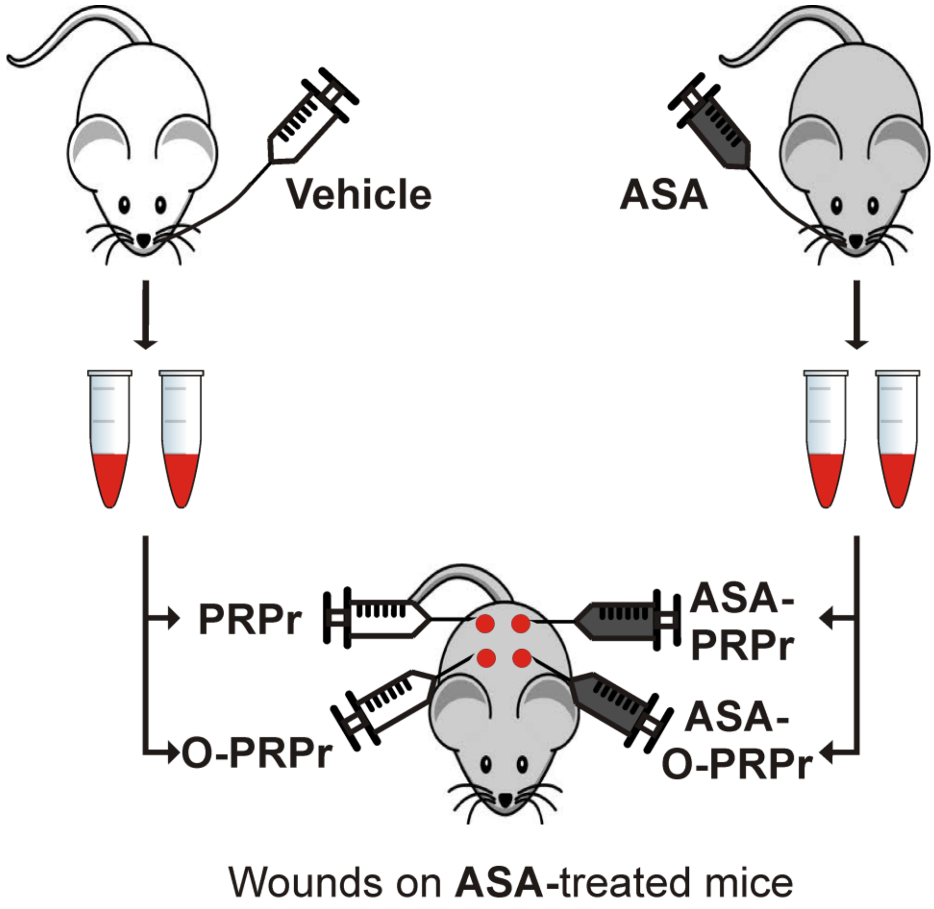

Supplemental Figure 5. Experimental design of wound. Mice were administered for 2 days with ASA (17.2 mg/Kg, equivalent to 100mg/day human dosage) or vehicle (saline) through oral gavage. PRPr or O-PRPr were obtained from ASA- or non-ASA-treated animals and injected in the periphery of wounds generated on the back skin of the ASA-treated mice. Drawing of mice, syringes and tubes were obtained from www.pixabay.com under the CC0 Creative Commons license.
